# Association of Muscle-Strengthening and Aerobic Physical Activity With Mortality in US Adults Aged 65 Years or Older

**DOI:** 10.1001/jamanetworkopen.2022.36778

**Published:** 2022-10-17

**Authors:** Bryant J. Webber, Katrina L. Piercy, Eric T. Hyde, Geoffrey P. Whitfield

**Affiliations:** 1 Division of Nutrition, Physical Activity, and Obesity, Centers for Disease Control and Prevention, Atlanta, Georgia; 2 Office of Disease Prevention and Health Promotion, US Department of Health and Human Services, Rockville, Maryland; 3 The Herbert Wertheim School of Public Health and Human Longevity Science, University of California, San Diego, La Jolla

## Abstract

This cohort study uses national data to explore the dose-response association between guideline-recommended physical activity and mortality in older adults.

## Introduction

The *Physical Activity Guidelines for Americans*, second edition, recommends that older adults (aged ≥65 years) participate in balance training, muscle-strengthening activities (MSAs; ≥2 days per week), and moderate to vigorous aerobic physical activity (MVPA; ≥150 minutes per week at moderate intensity, ≥75 minutes per week at vigorous intensity, or an equivalent combination).^1^ Evidence for MSAs in older adults is mostly based on fall prevention.^2^ This study explored the dose-response association between MSA and all-cause mortality in older adults, independent of and combined with MVPA, and characterized age-stratified associations.

## Methods

In this cohort study, we assessed self-reported leisure time physical activity and deaths among 1998-2018 National Health Interview Survey (NHIS) participants, using the 2019 linked NHIS and National Death Index mortality files.^3^ The NHIS is a nationally representative sample of the civilian, noninstitutionalized US population. The survey is approved by the National Center for Health Statistics, and all participants provide verbal consent.^4^ Between June 1 and July 5, 2022, we calculated weekly MVPA as the sum of moderate minutes and doubled vigorous minutes. In addition to the binary guidelines, we defined 4 levels of MSA by weekly episodes (0-1, 2-3, 4-6, and 7-28) and 4 levels of MVPA by weekly minutes (<10, 10-149, 150-300, and >300).^1^ We determined hazard ratios and 95% CIs for all-cause mortality using Cox regression, adjusting for sex; age; race and ethnicity; education; marital status; body mass index; smoking; alcohol consumption; and baseline presence of hypertension, heart disease, stroke, diabetes, cancer, chronic obstructive pulmonary disease, and asthma. We tested the proportional hazards assumption with Kaplan-Meier curves and Schoenfeld residuals and tested for interaction between MSA and MVPA on all-cause mortality. Of participants aged 65 years or older at the time of interview and eligible for National Death Index linkage (n = 131 418), we excluded those with incomplete data (n = 7827) and, to mitigate bias and confounding, those who died within 2 years of the interview (n = 8102). We used SAS, version 9.4 (SAS Institute Inc) statistical software to account for NHIS strata, clusters, and weights. This study followed STROBE reporting guidelines.

## Results

The 115 489 participants were predominantly women (70 451 [weighted 57.3%] vs 45 038 [42.7%] men), aged 65 to 74 years (64 322 [57.8%]), and White (86 404 [80.4%] vs 13 558 [8.4%] Black, 10 765 [7.3%] Hispanic, and 4762 [3.9%] other). During a mean follow-up of 7.9 years, 44 794 deaths occurred. No interaction was evident between MSA and MVPA categories. Adjusting for MVPA, 2 to 3 and 4 to 6 MSA episodes per week (but not 7 to 28 episodes per week) were associated with a lower hazard of all-cause mortality, compared with fewer than 2 episodes. Adjusting for MSA, 10 to 149, 150 to 300, and more than 300 MVPA minutes per week were associated with a lower hazard of all-cause mortality vs less than 10 minutes per week. Combinations of MSA and MVPA had lower hazard estimates (Table). Meeting both the strength and aerobic guidelines, vs meeting neither, was associated with a lower hazard of all-cause mortality among participants aged 65 to 85 years or older (Figure).

**Table.** Independent and Joint Associations Between Muscle Strengthening and Aerobic Physical Activity Levels and All-Cause Mortality in Older Adults^a^

Strength (MSA episodes/wk)	Aerobic (MVPA min/wk)	No. of participants	No. of deaths	Adjusted HR (95% CI)^b^
**Strength only**				
0-1	NA	99 298	40 502	1 [Reference]
2-3	NA	8206	1910	0.83 (0.79-0.87)
4-6	NA	2769	615	0.79 (0.72-0.86)
7-28	NA	5216	1767	0.98 (0.93-1.04)
**Aerobic only**				
NA	<10	54 421	26 033	1 [Reference]
NA	10-149	23 276	8136	0.83 (0.81-0.86)
NA	150-300	14 692	4577	0.75 (0.72-0.78)
NA	>300	23 100	6048	0.68 (0.66-0.71)
**Strength and aerobic**				
0-1	<10	52 525	25 219	1 [Reference]
2-3	<10	708	274	0.91 (0.80-1.05)
4-6	<10	214	86	0.76 (0.58-1.00)
7-28	<10	974	454	0.96 (0.84-1.09)
0-1	10-149	20 046	7140	0.83 (0.81-0.86)
2-3	10-149	1847	510	0.77 (0.70-0.85)
4-6	10-149	410	108	0.71 (0.59-0.86)
7-28	10-149	973	378	0.91 (0.81-1.03)
0-1	150-300	11 371	3728	0.76 (0.73-0.79)
2-3	150-300	1877	433	0.66 (0.59-0.73)
4-6	150-300	568	124	0.58 (0.49-0.68)
7-28	150-300	876	292	0.89 (0.80-1.00)
0-1	>300	15 356	4415	0.70 (0.67-0.72)
2-3	>300	3774	693	0.61 (0.56-0.67)
4-6	>300	1577	297	0.63 (0.56-0.72)
7-28	>300	2393	643	0.69 (0.63-0.75)

Abbreviations: HR, hazard ratio; MSA, muscle-strengthening activity; MVPA, moderate to vigorous physical activity; NA, not applicable.

^a^ Based on leisure time physical activity and covariate data from the National Health Interview Survey (1998-2018) linked to mortality in the National Death Index (1998-2019), excluding deaths within 2 years of the interview (n = 115 489).

^b^ All HRs adjusted for sex; age (continuous); self-reported race/ethnicity (Hispanic, non-Hispanic Black, non-Hispanic White, non-Hispanic other); education (less than high school, high school graduate, some college, college graduate or higher); marital status (currently married; divorced, separated, or widowed; never married; unknown); body mass index (continuous); smoking (never, former, current); alcohol consumption (never, former, current low, current high); and presence of hypertension, heart disease, stroke, diabetes, cancer, chronic obstructive pulmonary disease, and asthma. Strength HRs also adjusted for MVPA minutes per week. Aerobic HRs also adjusted for MSA episodes per week.

**Figure.**
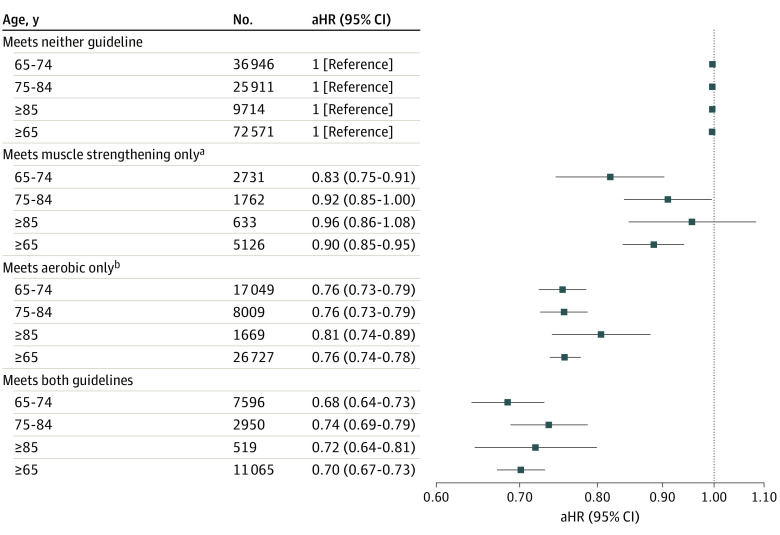
Associations Between Meeting Strength and Aerobic Physical Activity Guidelines and All-Cause Mortality by Age Group of Older Adults Hazard ratios are adjusted for sex; age (continuous); self-reported race and ethnicity (Hispanic, non-Hispanic Black, non-Hispanic White, non-Hispanic other); education (less than high school, high school graduate, some college, college graduate or higher); marital status (currently married; divorced, separated, or widowed; never married; unknown); body mass index (continuous); smoking (never, former, current); alcohol consumption (never, former, current but not heavy, current heavy [previous year average >7 drinks per week for women and >14 drinks per week for men]); and presence of heart disease, stroke, diabetes, cancer, chronic obstructive pulmonary disease, and asthma. Plotted on a logarithmic (base 10) scale. Based on the linked National Health Information Survey (1998-2018) and National Death Index (1998-2019). Deaths within 2 years of interview were excluded. Whiskers represent 95% CIs, and aHR indicates adjusted hazard ratio. ^a^Muscle strengthening activity of 2 episodes per week or more. ^b^Aerobic physical activity of 150 minutes per week or more at moderate intensity, 75 minutes per week or more at vigorous intensity, or an equivalent combination.

## Discussion

Leisure time MSA and MVPA were independently associated with lower all-cause mortality in this cohort study of US adults aged 65 years or older. By using finer age and physical activity categories, a larger sample, and longer follow-up, we build on earlier studies^5,6^ and offer new insights for older adults and their health care professionals. First, the U-shaped dose-response between MSA and mortality, independent of aerobic physical activity, suggests that 2 to 6 episodes per week may be optimal.^6^ Second, the age-stratified associations indicate that current physical activity guidelines are important for all older adults, including those aged 85 years or older. Limitations of these findings are possible unmeasured confounding and biases associated with self-reported physical activity data; nonetheless, this study highlights the mortality benefit of both MSA and MVPA for older adults of any age.
